# Functional coupling analysis suggests link between the obesity gene FTO and the BDNF-NTRK2 signaling pathway

**DOI:** 10.1186/1471-2202-12-117

**Published:** 2011-11-16

**Authors:** Mathias Rask-Andersen, Markus Sällman Almén, Hans R Olausen, Pawel K Olszewski, Jenny Eriksson, Rohit A Chavan, Allen S Levine, Robert Fredriksson, Helgi B Schiöth

**Affiliations:** 1 Department of Neuroscience, Functional Pharmacology, Uppsala University, BMC, Uppsala SE 75124, Sweden; 2 Minnesota Obesity Center, Department of Food Science and Nutrition, Saint Paul, MN 55108, USA

## Abstract

**Background:**

The Fat mass and obesity gene (FTO) has been identified through genome wide association studies as an important genetic factor contributing to a higher body mass index (BMI). However, the molecular context in which this effect is mediated has yet to be determined. We investigated the potential molecular network for FTO by analyzing co-expression and protein-protein interaction databases, Coxpresdb and IntAct, as well as the functional coupling predicting multi-source database, FunCoup. Hypothalamic expression of FTO-linked genes defined with this bioinformatics approach was subsequently studied using quantitative real time-PCR in mouse feeding models known to affect FTO expression.

**Results:**

We identified several candidate genes for functional coupling to FTO through database studies and selected nine for further study in animal models. We observed hypothalamic expression of Profilin 2 (Pfn2), cAMP-dependent protein kinase catalytic subunit beta (Prkacb), Brain derived neurotrophic factor (Bdnf), neurotrophic tyrosine kinase, receptor, type 2 (Ntrk2), Signal transducer and activator of transcription 3 (Stat3), and Btbd12 to be co-regulated in concert with Fto. Pfn2 and Prkacb have previously not been linked to feeding regulation.

**Conclusions:**

Gene expression studies validate several candidates generated through database studies of possible FTO-interactors. We speculate about a wider functional role for FTO in the context of current and recent findings, such as in extracellular ligand-induced neuronal plasticity via NTRK2/BDNF, possibly via interaction with the transcription factor CCAAT/enhancer binding protein β (C/EBPβ).

## Background

Genome-wide association studies consistently show that fat mass and obesity-related gene (FTO) gene polymorphism is the strongest known genetic factor associated with the development of higher BMI and obesity [1-5]. Several single nucleotide polymorphisms (SNPs) in this gene have been linked with elevated BMI in various populations [3,6-12]. Recent studies have shown that FTO risk alleles are preferentially transcribed in heterozygote individuals, and this is facilitated by increased binding of transcription factor CUX1 to the regulatory elements in FTO intron 1 [13-15]. Transgenic mice carrying additional copies of the Fto gene also show an Fto expression-dependent body weight response [16].

Data obtained in studies on Fto knockout and mutant mice (Fto^I367A^) suggest that FTO is involved in energy homeostasis, metabolism and adipogenesis [17]. Hypothalamic FTO is highly regulated in mouse and rat feeding models [18,19]. However, our knowledge of the molecular and physiological functions of FTO is still very limited. The FTO protein has some structural similarities to canonical demethylase enzymes and its demethylating activity has been demonstrated *in vitro *[20]. Recent data suggest a role for FTO in co-regulation of transcription. Using a reporter assay, Wu et al. were able to observe co-expression of FTO and CCAAT-enhancer binding protein β (C/EBPβ) to increase expression of a reporter gene driven by a C/EBP response element (CEBPRE). This element contained three CpG-sites for methylation and additional analysis showed coexpression of FTO and C/EBPβ to partially attenuate the reduced reporter gene expression induced by methylation of CEBPREs [21]. C/EBPβ can be termed an "early-response" transcription factor, as it links extracellular signaling pathways to gene regulation and long-term changes in the cell's differentiation. It has been associated with a wide range of functions, from adipogenesis and energy homeostasis control to immune responses, neuroprotection, and synaptic plasticity [22,23]. In line with that, dysregulation of FTO may lead not only to the impairment of energy homeostasis, but also to other abnormalities, including improper formation of neuronal networks. For example, a rare loss-of-function mutation documented in a Palestinian family showed that FTO deficiency leads to growth retardation and severe developmental defects of the central nervous system [24].

In order to determine a molecular mechanistic context for FTO, we utilized information from co-expression-, and protein-protein interaction databases and identified nine candidate genes for functional coupling to FTO. We then performed qRT-PCR expression analysis of murine hypothalami to detect whether the network candidate genes were co-regulated with FTO in a feeding restriction paradigm known to affect FTO expression in a differential manner. We found changes in the expression of Pfn2, Prkacb and Bdnf to parallel FTO mRNA upregulation during food deprivation. We also observed co-regulation of Fto and brain-derived neurotrophic factor (Bdnf) and its receptor TrkB (Ntrk2) in the sated state, and of Fto and Btbd12, Pfn2 and Stat3 in the food-deprived state.

## Methods

### Databases and gene interaction analysis

The FunCoup database (http://funcoup.sbc.su.se) predicts protein functional coupling through integration of data from a number of high-throughput functional assays. It uses information from 51 data sets containing mRNA and protein co-expression, protein-protein interaction, subcellular colocalization, phylogenetic profile similarity, shared transcription factor binding, shared miRNA targeting and domain associations. The database uses Bayesian statistics to predict the probability of functional coupling between two proteins and presents this as a probabilistic confidence value *pfc*) ranging from 0 to 1 [25]. The Coxpresdb (coexpressed gene database) (http://coxpresdb.jp) uses Affymetrix GeneChip array data (Human Genome U133 plus 2.0 array and Mouse Genome 430 2.0 array) and presents interactions between genes based on probe-to-probe expression pattern similarities and estimates the strength of the interaction by combining the ranks of co-expression for the corresponding gene pair [26]. The protein-protein interaction database IntAct (http://www.ebi.ac.uk/intact/main.xhtml) collects data from protein-protein interaction experiments, e.g. yeast two hybrid screenings and co-immunoprecipitation assays. The database allows submissions from independent research groups and currently encompasses data on approximately 200,000 protein-protein interactions in several species. IntAct also contains links to the publications describing the interactions [27]. Databases were queried in default settings with "FTO" as the search term. Human FTO interactions were queried in Funcoup and results for the human FTO gene were selected in the Coxpresdb database.

### Mouse feeding models

In the gene expression analysis, we utilized the tissue collected in the experiments described previously [19]. In short, male C57BL/6J mice (Scanbur, Sweden) were housed individually or in groups of two in macrolon cages with LD 12:12 (lights on at 07.00). Animals were twelve weeks old at the beginning of the experiment. Water and standard chow (Lactamin, Sweden) were available *ad libitum *unless specified otherwise. All animal procedures were approved by the Uppsala Animal Ethical Committee (ID: C228/7 & C262/7) and followed the guidelines of Swedish legislation on animal experimentation (Animal Welfare Act SFS1998:56) and European Union legislation (Convention ETS123 and Directive 86/609/EEC).

### 16-hr food deprivation

Chow was removed just before the onset of darkness and mice were sacrificed 16 hours later. Control mice had *ad libitum *access to chow. Each group contained 8 animals. Hypothalami were dissected and immersed in RNAlater.

### Ad libitum chow fed cohort

A larger group of *ad libitum *chow fed animals was used to better study co-regulation of FTO with other genes. 10 group-housed mice had ad libitum access to chow for 48 hours before sacrifice and dissection of hypothalami.

### Generation of cDNA and qRT-PCR

The tissue was kept in RNAlater at room temperature for 2 h and then stored at -80°C until further processing. Tissues were homogenized by sonification (Branson sonifier B 15) and RNA was purified from the samples using the TRIzol (Sigma-Aldrich, Sweden) method [28]. Samples were treated with Dnase I (Roche Diagnostics, Scandinavia) to remove residual DNA contamination. DNA contamination after Dnase I treatment was checked with PCR. Complementary DNA, cDNA, was then generated using reverse MLV reverse transcriptase (Invitrogen, Sweden) according to the manufacturer's specifications. PCR reactions were run in a total volume of 20 ul using Taq polymerase (Biotools, Sweden). Each reaction was performed in duplicate and contained 75 mM Tris/HCL, 50 mM KCl and 20 mM (NH_4)2_SO_4, _4 mM MgCl_2_, 0.25 mM dNTPs, 1:20 DMSO, 20 mU/ul Taq polymerase, 50 mM forward and reverse primer and 1:4 SYBR-green (Invitrogen, Sweden). Reactions were run on iCycler temperature cyclers and fluorescence was measured using MyiQ single color real time PCR detection system. Data were analyzed using iQ5 software (BioRad, Sweden). Gene expression was normalized between the samples using the geometric mean of the most stable housekeeping genes, in accordance with the geNorm-method as previously published by Vandesompele et al. [29].

### Statistical analysis

D'agostino & Pearson omnibus normality test was used to test normal distribution of gene expression data. Student's t-test was used to test the difference in gene expression between *ad libitum *chow fed, and food restricted mice. Linear regression was used to determine the relationship between expression of FTO and candidate genes. FTO was considered to correlate to candidate genes if the slope of the trendline significantly deviated from zero. For all tests nominal p-values < 0.05 were considered significant. The statistical analysis was performed with Prism v5.02 (GraphPad Software, San Diego, California, USA, http://www.graphpad.com).

## Results

### Database analysis suggests several FTO-interaction candidates

**Figure 1:**
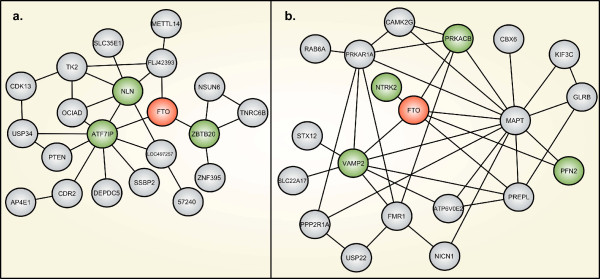
**Network images of FTO and candidates genes**. Coxpresdb (a) extracts expression data from affymetrix gene-chip arrays (human genome u133 plus 2.0 array and mouse genome 430 2.0 array) run on 3749 human and 2226 mouse samples [26]. The FunCoup database (b) integrates heterogenous data from 51 unique datasets, containing information on mRNA co-expression, protein-protein interaction, sub-cellular location etc., and uses Bayesian statistics to generate a probabilistic score for functional coupling between genes [25].

The FTO-interaction networks generated by Coxpresdb and FunCoup are presented in Figure 1 and genes selected for further analysis are presented in Table 1. A cut-off *pfc*-value of 0.4 was selected to remove the weakest interactions generated by the FunCoup database (see Figure 1a). The four genes with the highest *pfc*-score were then selected for analysis in animal models (Table 1). Candidate genes in close proximity to FTO in the network generated by Coxpresdb, were selected for further analysis. The FLJ42393-transcript was excluded from further analysis as it does not encode a functional protein according to Ensembl. The gene encoding neurolysin (NLN) was included instead due to its high connectivity and proximity to several candidate FTO interactors generated by this database (Figure 1a). We were unsuccessful in generating primers for the Aft7ip gene. The IntAct database generated only one candidate gene, SLX4 structure-specific endonuclease subunit homolog (BTBD12). This interaction was observed in an anti-tag co-immunoprecipitation experiment aimed at finding protein-protein interactions to BTBD12 [30].

**Table 1:** Candidates genes for FTO-interaction studies based on results from queries in Funcoup, Coxpresdb and IntAct (see Figure 1).

Candidate genes	Name	Function
PFN2	Profilin 2	Regulates actin polymerization in response to extracellular signals

NRTK2	Neurotrophic tyrosine kinase receptor type 2	Receptor for BDNF

BDNF	Brain derived neurotrophic factor	involved in neuronal plasticity & differentiation

PRKACB	cAMP dependent protein kinase catalytic subunit beta	Ser/Thr protein kinase, catalytic subunit of AMP-activated protein kinase (AMPK)

VAMP2	Vesicle-associated membrane protein 2. alt. Synaptobrevin-2	Involved in the targeting and/or fusion of transport vesicles to their target membrane.

STAT3	Signal transducer and activator of transcription 3	Transcription factor involved in cell growth and apoptosis. Responds to leptin signalling.

NLN	Neurolysin	Hydrolyzes oligopeptides such as neurotensin, bradykinin and dynorphin A

BTBD12	SLX4 structure-specific endonuclease subunit homolog	DNA-repair, endonuclease activity.

ZBTB20	Zinc finger and BTB domain-containing protein 20	May be a transcription factor that may be involved in hematopoiesis, oncogenesis, and immune responses

ATF7IP	Activating transcription factor 7-interacting protein	A regulator of telomerase reverse transcriptase (TERT) expression

STAT3 was included in our analysis due to previous publications suggesting a link between this gene and FTO [36].

### Feeding deprivation causes up-regulation of hypothalamic Fto, Pfn2 and Prkacb as well as down-regulation of Bdnf

**Figure 2:**
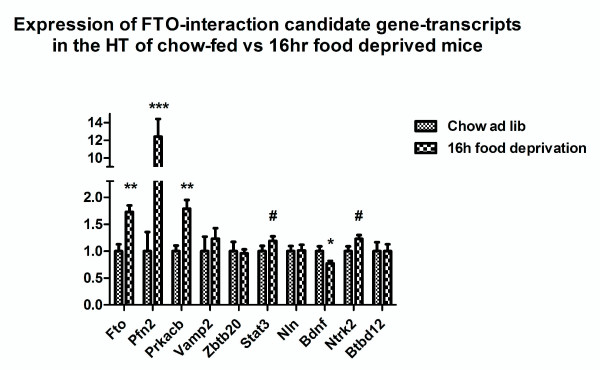
**mRNA expression levels in hypothalamus of 16 hour food deprived vs chow fed mice**. Asterisks denote significance of Student's t-test for difference of means. * = p < 0.05, ** = p < 0.005, *** = p < 0.001, # denotes trend towards significance, p < 0.10.

**Figure 3:**
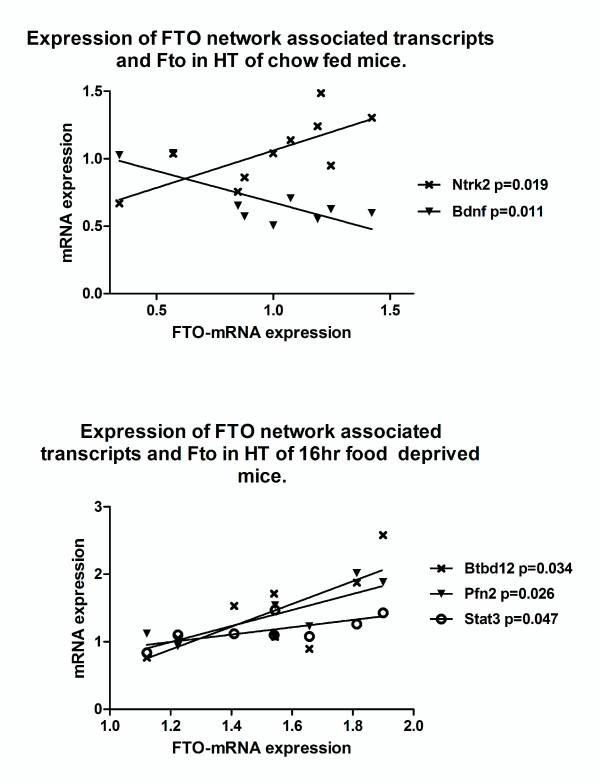
**Analysis of mRNA expression measured with quantitative RT-PCR**. (a) Hypothalamic expression of Ntrk2 and Bdnf correlates with Fto expression in 48 hour *ad libitum *chow fed mice. (b) Hypothalamic expression of Btbd12, Pfn2 and Stat3 correlates with Fto-expression in 16 hr food restricted mice, where Fto is upregulated (see figure 2).

Fto was significantly upregulated in the hypothalamus of food-deprived mice (p = 0.0012) in good agreement with previously published findings [18,19]. Several FTO network interaction candidates were also regulated in these mice (Figure 2). Pfn2 showed a twelve-fold increase in expression (p = 0.0001). Prkacb was up-regulated as well (p = 0.0017). Bdnf, the ligand for Ntrk2, was down-regulated in food-deprived mice (P = 0.04). Ntrk2 and Stat3 showed a trend toward up-regulation, however these differences did not reach statistical significance (p = 0.07 and p = 0.17, respectively). We also observe hypothalamic expression levels of Pfn2, Stat3 and Btbd12 to correlate with Fto-expression in food deprived mice but not in chow fed mice (see Figure 3).

### Hypothalamic FTO is co-regulated with Ntrk2 and BDNF in chow fed mice

In the cohort of 48 hour chow fed mice we observed the expression of FTO to show a higher degree of variation compared to chow fed animals in the 16 hour paradigm, which is most likely due to larger individual differences in food consumption. The variation in gene expression was shown to follow a normal distribution. Analysis also showed that Ntrk2-expression correlated positively to expression of Fto (p = 0.0105) while Bdnf correlated negatively (p = 0.0193) (see Figure 3 & Table 2).

**Table 2:** Correlation data for candidate genes.

Column1	Gene	Slope	95% Confidence Intervals	Goodness of Fit, R square	P value	n
*Ad libitum *	Pfn2	0.57	-0.1531 to 1.286	0.29	0.107	10

Chow fed	**Ntrk2**	**0.55**	**0.1165 to 0.9898**	**0.52**	**0.019**	**10**

Mice	**Bdnf**	**-0.47**	**-0.7881 to -0.1488**	**0.63**	**0.011**	**10**

	Prkacb	-0.06	-0.3902 to 0.2681	0.02	0.680	10

	*Vamp2*	*0.29*	-*2.319 to 2.905*	*0.01*	*0.793*	*8*

	*Stat3*	*0.50*	-*1.635 to 2.626*	*0.05*	*0.590*	*8*

	*Nln*	*0.63*	-*1.469 to 2.737*	*0.08*	*0.489*	*8*

	*Btbd12*	*2.38*	-*3.681 to 8.433*	*0.13*	*0.374*	*8*

	*Zbtb20*	*2.29*	-*1.129 to 5.710*	*0.31*	*0.152*	*8*

	Atf7ip	-	-	-	-	-

Food deprived	**pfn2**	**1.19**	**0.2082 to 2.171**	**0.66**	**0.026**	8

mice	Ntrk2	0.09	-0.54 to 0.73	0.03	0.724	8

	Bdnf	0.06	-52 to 0.64	0.02	0.792	8

	Prkacb	-0.04	-1.198 to 1.113	0.00	0.932	8

	Vamp2	0.71	-0.5620 to 1.983	0.24	0.221	8

	**Stat3**	**0.54**	**0.01046 to 1.078**	**0.51**	**0.047**	**8**

	Nln	0.71	-0.4190 to 1.832	0.28	0.175	8

	**Btbd12**	**1.69**	**0.1775 to 3.198**	**0.55**	**0.034**	**8**

	Zbtb20	0.65	-0.3582 to 1.664	0.29	0.165	8

	Atf7ip	-	-	-	-	-

The expression of genes in bold text are significantly correlated to expression of Fto (p < 0.05). Genes in cursive text were run on chow fed mice in the 16 hour food restriction paradigm. Hypothalamic expression of Ntrk2 and Bdnf correlate to Fto-expression positively and negatively, respectively, in *ad libitum *chow fed mice. Pfn2, Stat3 and Btbd12 were positively correlated to expression of Fto in hypothalami of 16 hour food restricted mice.

## Discussion

We identified nine candidate genes for functional coupling and interaction to FTO through database analysis and previous publications. In order to test the validity of this network, expression of network candidate genes was analyzed in a food deprivation model known to induce up-regulation of Fto. We confirmed increased levels of Fto mRNA and observed simultaneous up-regulation of four out of the nine genes in the hypothalamus during food deprivation: Pfn2, Prkacb, TrkB and Stat3; as well as down-regulation of Bdnf. Bdnf, Ntrk2 and Stat3 have been previously implicated in the regulation of energy homeostasis. Bdnf inhibits feeding and increases energy expenditure when injected into the ventromedial hypothalamus (VMH) and paraventricular nucleus of the hypothalamus (PVN) [31-34]. Stat3 is required for the anorexic effect of leptin-receptor signalling in the ARH [35]. Stat3 is also up-regulated in response to overexpression of AAV injection-induced Fto overexpression in the arcuate nucleus [36]. However, two of the genes, Prkacb and Pfn2, have not previously been implicated in any feeding/obesity paradigms. Intriguingly, we observed the expression of Fto to correlate with expression of Ntrk2 and Bdnf in the hypothalamus of chow-fed mice (p = 0.019 and 0.011, respectively) (Figure 3 & Table 2). We also observed a strong linearity between Fto expression and that of Ntrk2 and Bdnf (R^2 ^= 0.52 and 0.63, respectively) (Table 2).

The expression and co-regulation analysis provides additional evidence for a functional coupling of FTO to the network candidate genes and validates these databases in terms of co-expression [37,38]. From this viewpoint, FunCoup has provided the most valuable leads as the genes it reports as candidates are shown not only to be regulated during starvation in a similar way as Fto, but also follows the expression of Fto in a linear fashion.

**Figure 4:**
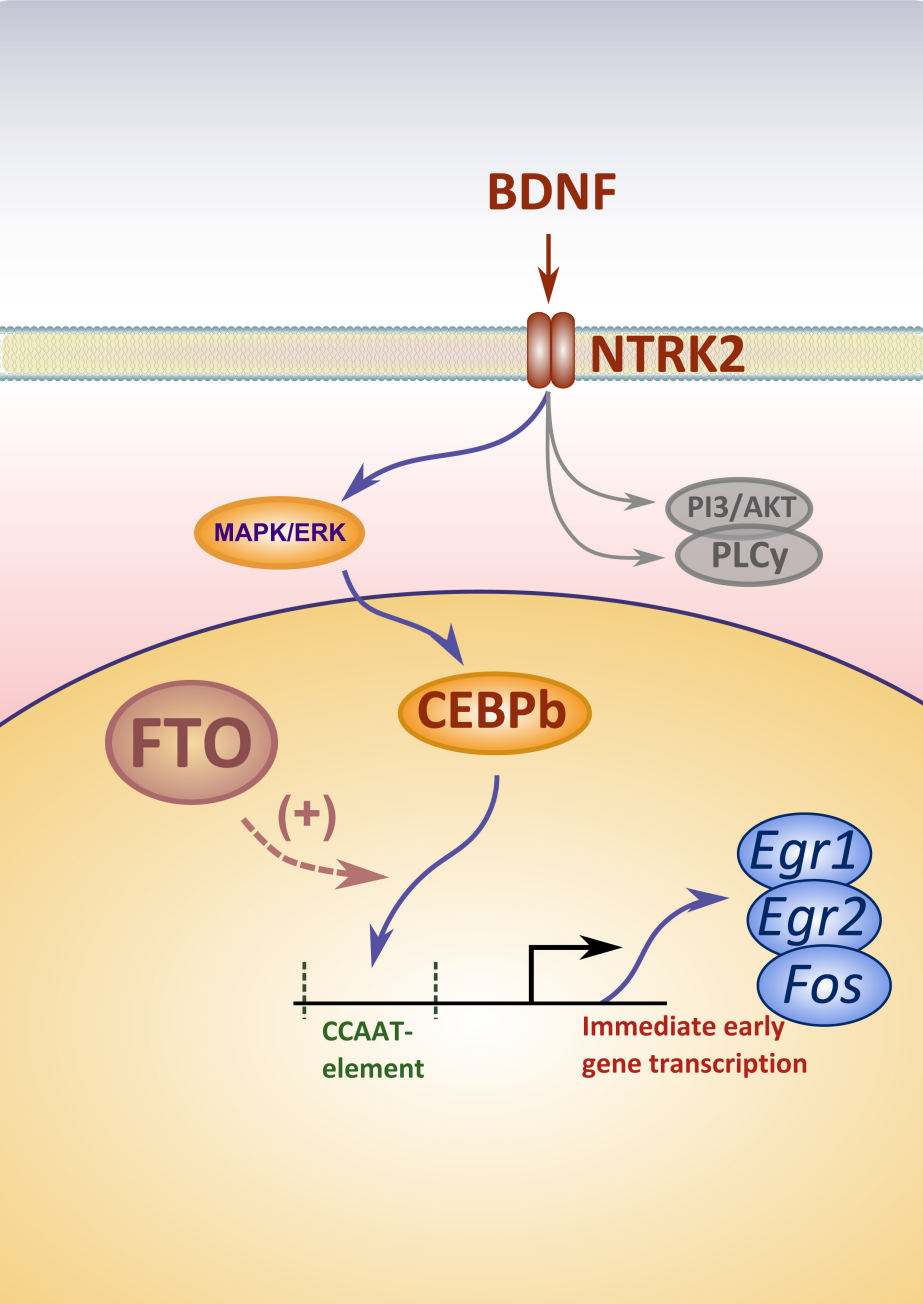
**In silico analysis and gene expression studies suggests functional coupling between FTO and the BDNF/NTRK2-signalling pathway**. Ablation of C/EBPs has been shown to reduce the expression of transcription factors Egr1, Egr2 (early growth response protein 1 and 2) and Fos (proto-oncogene c-Fos) following BDNF exposure in cultured neuronal cells [21,39] linking BDNF-NTRK2 to gene transcription via C/EBPs. C/EBPβ is a substrate for phosphorylation by MAPK, one of three downstream pathways of BDNF-NTRK2 signalling [49,50]. C/EBPs mediate the immediate early gene response of NTRK2-signalling. Recent findings by Wu et al. suggest FTO to attenuate epigenetic control of gene regulation via methylation of CpG-sites in C/EBPβ response elements (CEBPREs) [21]. In silico analysis, as well as expression studies, point to a functional coupling between FTO and the BDNF/NTRK2-signalling pathway, potentially mediated by the increased binding of C/EBPβ methylated response elements

FTO has been shown to exhibit de-methylase activity [20] with a putative role in epigenetic regulation of gene expression. However, a recent study found evidence that FTO increased binding of transcription factor CCAAT-enhancer binding protein beta (C/EBPβ) to methylated as well as de-methylated promoter sites. This study suggests a unique functional role for FTO: the enabling of transcription factors to bypass methylation specific epigenetic control of gene expression [21]. Also of great interest is that C/EBPβ has been reported to mediate the immediate early gene response induced by BDNF signaling via NTRK2 [39], by acting as an inducible transcription factor downstream of MAPK/ERK in the NTRK2 signaling pathway. We have prepared a hypothetical model for the involvement of FTO in the BDNF-NTRK2 signaling pathway, which integrates our data and previous findings on BDNF-NTRK2, FTO and C/EBPβ (Figure 4).

Based on our observations, we speculate that FTO may affect the regulation of ligand-induced neuronal plasticity via BDNF-NTRK2, PRKACB, STAT3 and PFN2. BDNF is involved in neuronal survivability, differentiation and formation of memory [40] and has also been associated to obesity in recent GWA-studies [41]. Pfn2-encoded profyllin 2 regulates important cellular functions such as endocytosis, vesicle recycling and actin assembly in CNS neurons [42]. Its effect on actin turnover may indicate a function in the regulation of neuronal plasticity in response to food intake. Neuronal plasticity occurs e.g. in the arcuate nucleus (ARH) in response to leptin signaling [43] and within the ARH - PVN pathways which develop during the perinatal period [44]. Interestingly, some FTO polymorphisms are associated with lower brain volumes in the frontal and occipital lobes [45]. As obesity itself is in some cases also associated with lower brain volume as well as cognitive deficits [46], FTO may be one of the genes bridging body weight dysregulation and other physiological abnormalities that oftentimes accompany abnormally low or high body weight. PRKACB (cAMP-dependant protein kinase catalytic subunit beta) encodes a catalytic subunit isoform of the protein kinase A holoenzyme (PKA). PRKACB phosphorylates the transcription factor, cAMP-response element-binding protein (CREB). CREB, in turn, regulates expression of several genes, including CEBPβ, BDNF, NTRK2, NPY and the glucocorticoid receptor. PRKACB has been linked to depression as lower expression of PRKACB in the prefrontal cortex occurs in major depression [47]. STAT3 is a transcription factor involved in cell growth, embryogenesis, apoptosis and cell motility. It is also one of the mediators of leptin signaling and regulates the expression of appetite regulators Agouti related peptide (AGRP) and pro-opiomelanocortin (POMC) [48]. This again suggests the potential role of Fto and its network of genes as a common molecular mechanism underlying co-regulation of body weight and processes related to cognition.

## Conclusions

We identified several candidate genes for functional coupling to FTO through database analysis and validated these by expression studies in relevant feeding models via qRT-PCR. Based on these findings, we hypothesized about the molecular context of FTO, discussing a putative involvement in neuronal plasticity by association to transcription factors downstream of ligand activated signaling pathways such as BDNF/NTRK2. These functional results provide new ideas for deducing the mechanisms affected by obesity related FTO-risk alleles.

## Authors' contributions

MRA performed the statistical analysis and drafted the manuscript. MSA conceived the study, performed the in silico analysis and participated in the design of the study. HRO and RAC performed the experiments and preliminary analysis. HRO also participated in drafting the manuscript. PKO and JE performed and designed the animal experiments. ASL and RF designed and conceived the study. HBS designed and conceived the study and drafted the manuscript. All authors have read and approved the final verison of the manuscript.
